# Case Report: Bioresorbable implant in anterior abdominal wall reconstruction in women of reproductive age. Case Report with pregnancy and a 10-year follow-up

**DOI:** 10.3389/fsurg.2026.1776962

**Published:** 2026-05-28

**Authors:** Michał Putko, Mateusz Zamkowski, Maciej Śmietański

**Affiliations:** 1 Śmietański Hernia Center, LUX MED Hospital Gdańsk, Gdańsk, Poland; 2 2nd Department of Radiology, Medical University of Gdańsk, Gdańsk, Poland

**Keywords:** abdominal hernia, absorbable implant, hernia repair, hernia repair in women of reproductive age, rectus abdominis diastasis

## Abstract

**Introduction:**

Surgical management of anterior abdominal wall hernias in women of reproductive age remains challenging, as repair techniques must accommodate physiological changes during pregnancy. While permanent synthetic meshes are effective, concerns exist regarding their mechanical behavior in the context of pregnancy in this patient population. Bioresorbable implants have emerged as a potential alternative; however, clinical data on pregnancy outcomes remain limited.

**Aim:**

To present a case of abdominal wall reconstruction using a bioresorbable poly-4-hydroxybutyrate (P4HB, Phasix®) mesh with subsequent pregnancy and long-term follow-up.

**Case description:**

A 42-year-old woman with a history of recurrent linea alba hernia and diastasis underwent retromuscular repair using a P4HB mesh. Sixteen months later, she conceived. Pregnancy and delivery were uneventful, with no abdominal wall-related symptoms. At 10-year follow-up, ultrasonography demonstrated no hernia recurrence, and laparoscopic inspection confirmed complete mesh resorption with preserved integrity of the linea alba.

**Conclusions:**

In this case, the use of a bioresorbable P4HB mesh was associated with an uncomplicated pregnancy and durable long-term outcome. However, given the single-case design and small defect size, these findings should be interpreted with caution and require confirmation in larger studies.

## Introduction

Anterior abdominal wall hernias are a common clinical problem and frequently require surgical intervention. In women of reproductive age, the choice of abdominal wall reconstruction technique requires particular caution, as the implanted material must accommodate the profound physiological changes occurring during pregnancy.

**Table 1:** Timeline of clinical events.

Time point	Clinical event
Year 0	Retromuscular repair of recurrent linea alba hernia with P4HB mesh
+16 months	Pregnancy
+25 months	Caesarean section (breech presentation)
+5 years	Diagnosis of bilateral inguinal hernias
+10 years	Laparoscopic TAPP repair; confirmation of complete mesh resorption

Permanent synthetic meshes, particularly polypropylene, are effective in preventing hernia recurrence but may exhibit mechanical properties that differ from native abdominal wall tissues. This mismatch may theoretically influence abdominal wall compliance during pregnancy. However, many patients with permanent mesh repairs undergo pregnancy without major complications, and the optimal approach in women planning pregnancy remains a subject of ongoing debate.

The introduction of bioresorbable materials, such as Phasix® (poly-4-hydroxybutyrate, P4HB), has opened a new perspective in hernia surgery. This implant undergoes gradual resorption over a period of 12–18 months while maintaining adequate mechanical strength during this time. Ultimately, it is fully resorbed, leaving reinforced native tissues. Although the literature reports its effectiveness in hernia repair, little is known about the behaviour of the abdominal wall following implant resorption in women who subsequently become pregnant.

The aim of the present study is to describe a case of a patient who underwent implantation of a bioresorbable Phasix® mesh, followed by observation of the natural behaviour of the linea alba and abdominal wall during pregnancy and over a 10-year follow-up period.

## Case description

### Diagnostic assesment

A 28-year-old female patient underwent surgery for a primary linea alba hernia. Primary suture repair of the hernia defect was performed; however, early recurrence occurred within a short period of time. Five years later, the patient was scheduled for surgical treatment due to recurrent hernia. The defect was intermittently irreducible, with a transverse hernia orifice measuring 3 cm and a hernia sac diameter of 8 cm (abdominal wall hernia classified as r1M2W1 according to the EHS classification).

### Therapeutic intervention

The procedure was performed using an open approach with implantation of a bioresorbable Phasix® mesh (poly-4-hydroxybutyrate, P4HB) measuring 16 × 10 cm, placed in the retromuscular space. Reconstruction of the linea alba was achieved using a continuous “small bites” suture technique. The intraoperative course was uneventful. The patient was discharged home in good general condition, with no signs of wound infection or local complications. The postoperative period was free from pain or wound-healing disorders.

### Follow-up and outcomes

Sixteen months after surgery, the patient became pregnant. The entire course of pregnancy was physiological, without excessive pain or impairment of abdominal wall function. During pregnancy, widening of the linea alba occurred but remained within the range of normal gestational changes. Delivery was completed by caesarean section due to breech presentation (Table 1).

Five years after the procedure, bilateral inguinal hernias were diagnosed. After a further five years, laparoscopic repair was performed, revealing bilateral L2 inguinal hernias according to the EHS classification, and a transabdominal preperitoneal (TAPP) repair was carried out. During laparoscopy, no evidence of hernia recurrence or diastasis of the linea alba was observed, and the implant was not identified, indicating complete resorption.

Throughout the 10-year follow-up period, no hernia recurrence was detected. Follow-up ultrasonography revealed no signs of linea alba diastasis. At the most recent ultrasound examination (10 years postoperatively), the linea alba measured approximately 2 cm in width, with no bulging, hernias, or signs of scar stretching. The patient remains asymptomatic and reports no abdominal wall-related complaints.

Ultrasonographic measurements were performed in a standardized supine position using a linear transducer, with assessment of the linea alba along the midline. Although examinations were part of routine follow-up rather than a formal study protocol, efforts were made to ensure consistency in measurement technique.

## Discussion

The presented case suggests that the use of a bioresorbable Phasix® implant may allow preservation of the natural physiology of the abdominal wall in a woman of reproductive age. During pregnancy, widening of the linea alba occurred; however, this process followed a typical physiological pattern and was not restricted by the presence of the implant. No local complications or chronic pain were observed. As Phasix® does not leave a permanent foreign body, it may reduce the risk of chronic pain, inflammation, and immunological reactions (1, 2). Prospective and observational studies have shown that Phasix® is associated with low hernia recurrence rates comparable to those observed with permanent synthetic meshes. In an animal model study by Novitsky et al. (3), as well as in a clinical analysis by Rosen et al. (1), favourable tissue healing outcomes without recurrence were reported over a 5-year follow-up period. Further confirmation was provided by studies by Itani et al. (2) and Deeken et al. (4), which demonstrated a low complication rate and satisfactory biomechanical properties of the mesh. However, available studies suggest that overall complication rates may be comparable to those observed with permanent synthetic meshes, and clear superiority has not been definitively established.

The literature regarding the direct use of Phasix® in pregnant women remains limited. Nevertheless, due to its bioresorbable nature and elasticity, this mesh may be well suited to the dynamic physiological changes occurring during pregnancy. Preclinical data suggest good adaptation of the mesh to soft tissues and low immunogenicity (5). In a published case report by Tulloh et al. (6), a woman who underwent hernia repair with Phasix® experienced an uncomplicated pregnancy and vaginal delivery.

Compared with non-absorbable synthetic meshes, Phasix® is associated with a lower incidence of chronic complications, such as persistent pain or fistula formation (1, 4). Owing to these properties, the mesh may represent a potential option in selected women planning pregnancy, for whom preservation of abdominal wall elasticity and avoidance of chronic symptoms are essential. This observation supports the notion that the implant does not interfere with the natural physiological changes of the abdominal wall during pregnancy.

The use of permanent polypropylene (PP) meshes in women planning pregnancy remains a matter of ongoing discussion. These meshes are effective and widely used in hernia repair, with well-established long-term outcomes. However, their mechanical properties differ from those of native abdominal wall tissues, which may theoretically influence abdominal wall compliance during pregnancy. Despite these considerations, many patients with prior mesh repair undergo pregnancy without major complications, and the optimal surgical approach in women of reproductive age remains debated in the literature (7–9).

Despite the lack of large randomised studies evaluating the safety and efficacy of resorbable poly-4-hydroxybutyrate (P4HB, Phasix®) meshes in women who are pregnant or planning pregnancy, the available clinical evidence suggests a favourable biocompatibility profile for this material. In the cases reported to date, the implant has been well tolerated, without pain-related symptoms or mechanical complications, and its presence has not adversely affected the course of pregnancy (10, 11). Owing to its high elasticity and gradual bioresorption, P4HB may allow preservation of the physiological compliance of the abdominal wall and appropriate adaptation of the linea alba during tissue stretching in pregnancy (5). Following complete degradation of the material, the abdominal wall demonstrates properties comparable to native tissue, without signs of excessive tension or deformation. Nevertheless, to definitively confirm the long-term safety and durability of reconstructive outcomes in this patient population, further prospective studies involving larger cohorts are required (12, 13).

This study has several limitations. First, it represents a single-case observation, which limits external validity. Second, no quantitative biomechanical measurements were performed, and conclusions regarding abdominal wall elasticity are based on indirect clinical and imaging findings. Third, ultrasonographic assessments were not conducted within a standardized research protocol, which may affect measurement reproducibility. Therefore, the findings should be considered hypothesis-generating.

It should be emphasized that the hernia defect in the present case was relatively small (3 cm), which is associated with a low baseline risk of recurrence regardless of the repair technique. Therefore, the favorable outcome observed in this case may not be directly generalizable to larger or more complex defects, where long-term mechanical reinforcement plays a more critical role.

An important consideration is the higher cost of bioresorbable P4HB meshes compared to permanent synthetic alternatives. Although their biological and mechanical properties may offer potential advantages in selected patients, the cost-effectiveness of their routine use remains uncertain and should be evaluated in the context of individual clinical scenarios and healthcare system constraints.

Despite promising characteristics, bioresorbable meshes are associated with uncertainties, including variability in degradation profiles and limited long-term comparative data. Careful patient selection remains essential.

## Take-home message

Bioresorbable P4HB mesh may represent a promising option in selected patients; however, further evidence is required before broader recommendations can be made. Its use may allow physiological adaptation of the abdominal wall during pregnancy while maintaining long-term durability

## Patient perspective

The patient reported high satisfaction with the treatment outcome. She experienced no abdominal wall-related symptoms during pregnancy or long-term follow-up and expressed confidence in her physical function and quality of life after surgery.

## Data Availability

The original contributions presented in the study are included in the article/Supplementary Material, further inquiries can be directed to the corresponding author.
